# Achieving consensus on quality indicators (QI) for pediatric Systemic Lupus Erythematosus (pSLE)

**DOI:** 10.1186/1546-0096-9-S1-P247

**Published:** 2011-09-14

**Authors:** Joshua Pendl, Matt Hollander, Shannen Nelson, Wajeeha Yousaf, Nicola Ruperto, Michael Beresford, Marisa Klein-Gitelman, Marilynn Punaro, Anne Stevens, Tadej Avcin, Graciela Espada, Tsz-Leung Lee, Yu-Lung Lau, Jennifer Huggins, Esi Morgan-DeWitt, Hermine I Brunner

**Affiliations:** 1 Cincinnati Children’s Hospital Medical Center, Cincinnati, OH; 2 Seattle Children’s, Seattle, WA; 3 University of Cincinnati, Cincinnati, OH; 4 G. Gaslini Research Institute, Genoa, Italy; 5 Royal Liverpool Children’s, Liverpool, UK; 6 Children’s Memorial Hospital, Chicago, IL; 7 University of Texas Southwestern Medical Center, Dallas, TX; 8 The Hospital for Sick Children, Toronto, Canada; 9 Children’s Hospital Ricardo Gutierrez, Buenos Aires, Argentina; 10 The University of Hong Kong, Hong Kong

## Background

QI are retrospectively measurable elements of practice performance for which there is evidence or consensus that can be used to assess the quality of care provided.

## Aim

To develop a set of consensus-derived QI for pSLE to serve as international benchmarks for the quality of patient care.

## Methods

Based on the medical literature a Delphi survey was created and distributed to the physician membership of PRES, PANLAR, CARRA and the ACR via e-mail. Consensus was considered 80% or higher.

## Results

There was consensus (97%) among the 297 respondents that simply applying QI developed by the ACR and EULAR for adults with SLE (adult QI) was insufficient and that distinct QI for pSLE were needed. Respondents concurred that 5 of the 20 ACR and 6 of the 24 EULAR adult QI are also suitable for pSLE. An additional 14 ACR and 13 EULAR adult QI might be useful for pSLE after modifications. There was no consensus whether to consider “Pregnancy” (45%) and “Reproductive Health” (65%) as domains in the set of pSLE QI.

## Conclusion

There is great demand among pediatric rheumatologists to develop QI for pSLE. Initial agreement has been reached about the types and domains of QI for pSLE, but additional discussion and consensus formation under consideration of the medical evidence is needed to finalize a set of QI for pSLE that can be used to define standard of care treatment for children and adolescents with pSLE.

